# Free triiodothyronine and triglyceride-glucose index interaction on metabolic dysfunction-associated steatotic liver disease risk in euthyroid individuals

**DOI:** 10.3389/fendo.2025.1526198

**Published:** 2025-04-24

**Authors:** Lei Gao, Wenxia Cui, Fenghui Pan, Dinghuang Mu, Weihong Zhou, Yun Hu

**Affiliations:** ^1^ Department of Geriatrics, Nanjing Drum Tower Hospital Clinical College of Nanjing Medical University, Nanjing, China; ^2^ Department of Geriatrics, Nanjing Drum Tower Hospital Clinical College of Nanjing University of Chinese Medicine, Nanjing, China; ^3^ Department of Health Management Center, Nanjing Drum Tower Hospital Clinical College of Nanjing Medical University, Nanjing, China; ^4^ Department of Chemistry, State Key Laboratory of Analytical Chemistry for Life Science, Nanjing University, Nanjing, China

**Keywords:** free triiodothyronine, triglyceride-glucose index index index, metabolic dysfunction-associated fatty liver disease, cross-sectional study, additive interaction

## Abstract

**Background:**

The link between thyroid function and insulin resistance and metabolic dysfunction-associated fatty liver disease (MASLD) is becoming increasingly recognized. The primary goal of this study was to investigate the relationship between free triiodothyronine (FT3) levels, the triglyceride-glucose index (TyG) index, and the likelihood of MASLD in euthyroid individuals.

**Methods:**

A cross-sectional analysis of 18,298 euthyroid individuals was conducted, comparing 6,144 with MASLD to 12,154 controls. The study evaluated indicators related to clinical, metabolic, and thyroid function. The combined effect of the FT3 and TyG index on the likelihood of MASLD was assessed using logistic regression.

**Results:**

The MASLD group presented with higher male prevalence, older age, and increased rates of hypertension and diabetes. Significant correlations were observed between FT3, TyG, and metabolic parameters. After controlling for potential confounders, FT3 remained significantly associated with increased MASLD risk (adjusted OR = 1.35, 95% CI: 1.23-1.49; *P* < 0.001). Similarly, the TyG index was independently associated with higher MASLD risk (adjusted OR = 3.99, 95% CI: 3.40-4.68; *P* < 0.001). The high FT3 (≥ 4.98 pmol/L)/high TyG (≥ 8.55) group exhibited significantly elevated MASLD risk compared to the low FT3/low TyG group (OR = 5.38, 95% CI: 4.62-6.26; *P* < 0.001).

**Conclusion:**

Elevated FT3 and TyG index are independently associated with an increased risk of MASLD, and they exhibit a significant synergistic additive interaction.

## Introduction

A new nomenclature called metabolic dysfunction-associated steatotic liver disease (MASLD) has been suggested to replace the term non-alcoholic fatty liver disease (NAFLD) (1). This change is made to better highlight the significant role of metabolic variables in the development of this disease (1). This change reflects the growing recognition that MASLD is not merely an isolated hepatic disorder but rather a complex manifestation of systemic metabolic derangements (2).

MASLD arises from a multifaceted interplay of factors, with genetic predisposition, insulin resistance, lipotoxicity, oxidative stress, and gut dysbiosis implicated as key contributors (3–7). These factors often act in concert, creating a vicious cycle that drives the initiation and progression of MASLD. The global prevalence of MASLD is alarming, affecting an estimated 30% of adults worldwide (8). Due to the high incidence of the condition, as well as the fact that it has the potential to advance to more serious liver disorders such as metabolic dysfunction-associated steatohepatitis (MASH), cirrhosis, and carcinoma of the liver, MASLD is recognized as a significant public health concern (8).

The thyroid hormones play a pivotal role in controlling the resting metabolic rate, influencing both the quantity of energy expended and the metabolic pathways utilized in the body, directly impacting hepatic lipid metabolism through regulation of fatty acid oxidation, synthesis, and lipoprotein secretion (9–11). Beyond lipid metabolism, thyroid hormones critically regulate hepatic glucose homeostasis through multiple mechanisms. Physiologically, thyroid hormones activate hepatic TRα/TRβ receptors, upregulating gluconeogenic enzymes (PEPCK, G6Pase) to enhance glucose production (12). They also stimulate glycogenolysis for rapid energy release (13). Importantly, excess triiodothyronine impairs insulin signaling by inhibiting IRS-1 phosphorylation and PI3K/Akt activation, promoting insulin resistance (14). There is a well-established link between hypothyroidism and the development and progression of MASLD (15). Furthermore, a significant correlation has been established between thyroid hormones and insulin resistance, obesity, and metabolic syndrome, according to a number of study findings (16–18). Free triiodothyronine (FT3), a biologically active thyroid hormone, has garnered interest. Research on free FT3 and NAFLD has revealed a robust, favorable connection in people with normal thyroid function, suggesting a role for FT3 in hepatic lipid metabolism and systemic energy balance (19–21). However, the precise mechanisms by which FT3 contributes to MASLD, particularly within the context of euthyroid individuals, remain poorly understood.

A convenient and reliable proxy for insulin resistance, the triglyceride-glucose index (TyG) index has been more used in recent years (22, 23). Produced from glucose and fasting triglyceride levels, this index is a novel biomarker. The TyG index offers a more practical and cost-effective alternative to the classical homeostasis model assessment of insulin resistance (such as homeostatic model assessment of insulin resistance (HOMA-IR)), which requires additional measurement of fasting insulin levels, while maintaining accuracy comparable to the conventional method. Given the established link between insulin resistance and MASLD, it is imperative that additional research into the potential link between the TyG index and MASLD.

The aim of this study was to investigate the independent associations of FT3 and the TyG index with MASLD, along with their potential additive interaction. Understanding the independent and synergistic effects of FT3 (active thyroid hormone) and the TyG index (insulin resistance indicator) on MASLD risk is essential for elucidating disease mechanisms and risk stratification. This study aims to further our comprehension of the hormonal modulation of MASLD, potentially paving the way for novel diagnostic and therapeutic strategies for this increasingly prevalent metabolic disease.

## Methods

### Subjects

**Figure 1:**
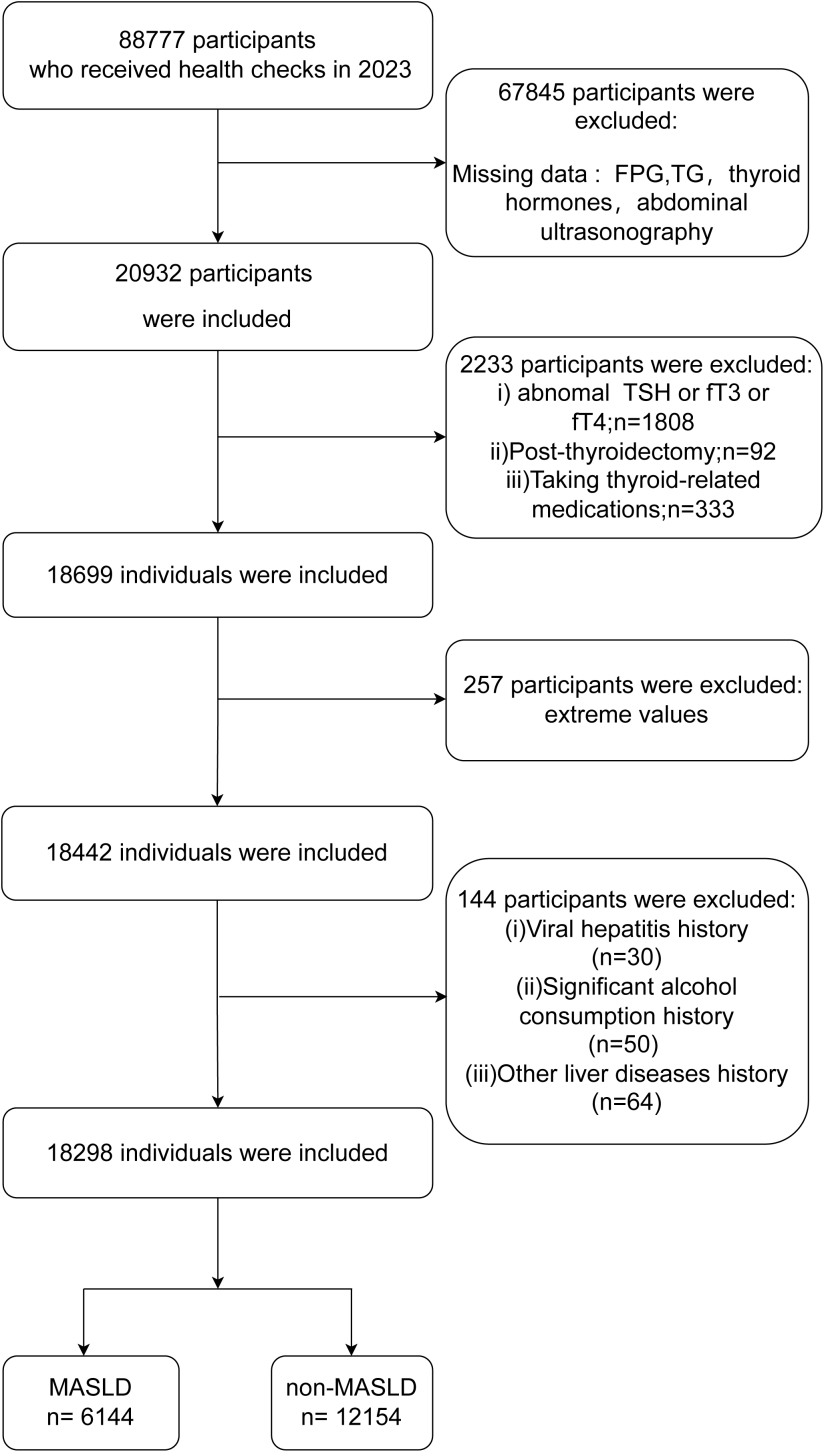
Flow chart of this study. MASLD, metabolic dysfunction-associated steatotic liver disease; FPG, fasting plasma glucose; TSH, thyroid-stimulating hormone; FT3, free triiodothyronine; FT4, free thyroxine.

The population of this study consisted of 88,777 individuals who were examined for their health at the Health Management Center of Nanjing Drum Tower Hospital in the year 2023. According to the following criteria, these individuals were not included in the study: individuals who lacked data on blood glucose, blood lipids, thyroid function, and abdominal ultrasound; individuals who had abnormal thyroid function (thyroid-stimulating hormone (TSH) > 4.2 pmol/L or < 0.27 pmol/L; FT3 > 6.8 pmol/L or < 3.1 pmol/L; free thyroxine (FT4) > 22 pmol/L or < 12 pmol/L); individuals who had undergone thyroid surgery or were taking medications related to the thyroid gland; individuals who possessed abnormal laboratory values; heavy alcohol consumers (daily alcohol intake > 20 g for female and > 30 g for male); patients with viral hepatitis; and individuals who had other chronic liver diseases. Within the scope of this investigation, a total of 18,298 individuals were involved. The flowchart of the study can be seen in **Figure 1**.

All procedures were performed in compliance with relevant laws and institutional guidelines and have been approved by the institutional committee (NO.2022-698-01) from the Nanjing Drum Tower Hospital Ethics Committee. Written informed consent was obtained from all individuals, and the study followed the ethical criteria of the Declaration of Helsinki.

### Data collection

We gathered information on the individuals’ age, sex, medications they were taking, and lifestyle activities such as smoking and drinking alcohol. Height, weight, blood pressure, and the waist circumferenc (WC) were all acquired through the process of measurement. To calculate body mass index (BMI), weight in kilograms was divided by the square of height in meters.

Individuals fasted overnight, avoiding alcohol and food, before blood samples were collected via peripheral venous puncture the following morning. Standardized procedures were used to measure blood biochemical indices (Beckman AU5400; Beckman Coulter Corp), total blood cell counts (Sysmex XN-1000; Sysmex Corp), and thyroid function indicators (Dxl 800; Beckman Coulter Corp). The TyG index was calculated using the formula: Ln[fasting triglycerides (TG)(mg/dL) × fasting plasma glucose (FPG)(mg/dL)/2].

### Diagnosis of hepatic steatosis by ultrasonography

Hepatic steatosis was diagnosed using abdominal ultrasonography, performed by two dedicated and trained physicians. The ultrasonographic criteria for hepatic steatosis included the following features: 1. Increased liver echogenicity (liver echogenicity higher than that of the spleen and kidney). 2. Attenuation of the ultrasound signal in the deep liver parenchyma (decreased echogenicity in the far field). 3. Poor visualization of intrahepatic vessel borders and diaphragm. The diagnosis of hepatic steatosis was established if the first criterion (increased liver echogenicity) was met, along with either the second or third criterion.

### Diagnostic criteria of MASLD

The diagnostic criteria for MASLD included the utilization of ultrasonography for the purpose of detecting hepatic steatosis, the presence of one or more cardio-metabolic risk factors, and the absence of a history of significant alcohol consumption or other chronic liver disorders. Individuals were excluded from the study if hepatic steatosis was detected but no cardio-metabolic risk factors (1).

Cardio-metabolic risk factors included: 1. BMI ≥ 23 kg/m², or WC > 94 cm (Male) or > 80 cm (Female); 2. Blood pressure ≥ 130/85 mmHg or specific anti-hypertensive drug treatment; 3. FPG ≥ 5.6 mmol/L or 2-hour post-load glucose ≥ 7.8 mmol/L or hemoglobin A1c (HbA1c) ≥ 5.7% or type 2 diabetes or treatment for type 2 diabetes; 4. Plasma high-density lipoprotein (HDL) ≤ 1.0 mmol/L (Male) or ≤ 1.3 mmol/L (Female) or lipid-lowering treatment; 5. TG ≥ 1.70 mmol/L or lipid lowering treatment (1).

### Statistical analysis

Data distribution was assessed using the Kolmogorov-Smirnov test. Continuous variables with normal distribution were described using mean and standard deviation, while non-normally distributed variables were summarized using median and interquartile range (IQR). Categorical variables were described using frequencies and percentages. Continuous variables were compared using the independent samples t-test (for normally distributed data) or the Mann-Whitney U test (for non-normally distributed data). Categorical variables were compared using the chi-square test. A *P*-value <0.05 was considered statistically significant.

The *TableSubgroupMultiGLM* function from the *jstable* R-package was applied to evaluate the association between MASLD and FT3 across different subgroups, and interaction *P*-values across different subgroups were estimated using the maximum likelihood ratio method (24). A *P*-value < 0.05 was considered to indicate a statistically significant interaction between subgroups.

Receiver Operating Characteristic (ROC) curve analysis determined optimal FT3 and TyG index cutoffs for MASLD prediction based on the maximum Youden index. The calculated cutoff values were 8.55 for the TyG index and 4.98 for FT3. Based on these cutoffs, participants were categorized into four groups: low FT3 and low TyG, high FT3 and low TyG, low FT3 and high TyG, and high FT3 and high TyG.

The *interactionR* R-package was used to calculate additive interactions between FT3 and the TyG index in relation to their impact on MASLD, and the relative excess risk due to interaction (RERI), attributable proportion (AP), and synergy index (SI) were estimated (25). A statistically significant additive interaction was considered to exist if RERI and AP > 0 and SI > 1 (26, 27).

All analyses were performed using R (version 4.3.1; https://www.R-project.org).

## Results

### Evaluation of MASLD and non-MASLD features in comparison

**Table 1** displays the features of individuals categorized into MASLD and non-MASLD groups. The analysis comprised 18,298 individuals, of whom 6,144 had MASLD and 12,154 did not. The results show significant variations across groups for many characteristics. The MASLD group had a greater male percentage (80.86%) than the non-MASLD group (48.80%), *P* < 0.001. In addition, individuals with MASLD had higher mean ages (48.32 ± 12.91 vs. 43.59 ± 13.67) and greater prevalence of hypertension (37.11% vs. 13.81%) and diabetes (15.67% vs. 4.69%) compared to those without MASLD (all *P* < 0.001). Individuals with MASLD had significantly higher WC, BMI, hemoglobin (HB), blood pressure, liver enzymes, FPG, uric acid (UA), and lipid levels, and significantly lower HDL levels compared to those without MASLD (all *P* < 0.001).

**Table 1:** Comparison of the characteristics between MASLD and non-MASLD ( $\bar{x}$ ± *s or M*[*IQR*] *or n(%)*).

Characteristic	Total (n=18298)	non-MASLD (n=12154)	MASLD (n=6144)	*P* value
Male, n (%)	10899 (59.56)	5931 (48.80)	4968 (80.86)	<0.001
Age (years)	45.18 ± 13.61	43.59 ± 13.67	48.32 ± 12.91	<0.001
Hypertension, n (%)	3959 (21.64)	1679 (13.81)	2280 (37.11)	<0.001
Diabetes, n (%)	1533 (8.38)	570 (4.69)	963 (15.67)	<0.001
BMI (Kg/m^2^)	24.41 ± 3.52	22.96 ± 2.78	27.26 ± 3.03	<0.001
WC (cm)	82.09 ± 11.40	77.30 ± 9.50	91.58 ± 8.53	<0.001
SBP (mmHg)	128.09 ± 17.85	119.56 ± 15.88	125.31 ± 16.50	<0.001
DBP (mmHg)	79.61 ± 11.64	74.44 ± 10.19	78.22 ± 10.92	<0.001
HB (g/L)	147.34 ± 14.90	143.72 ± 14.81	154.50 ± 12.27	<0.001
ALT (IU/L)	19.30[13.70, 29.30]	15.90[12.00, 21.80]	30.60[22.10, 44.80]	<0.001
AST (IU/L)	19.50 [16.50, 23.70]	18.30 [15.70, 21.60]	22.80[19.10, 28.50]	<0.001
GGT (IU/L)	23.80[16.60, 37.60]	19.30[14.70, 27.50]	37.50[26.90, 57.00]	<0.001
FPG (mmol/L)	5.20 ± 0.96	5.00 ± 0.76	5.60 ± 1.16	<0.001
UA (umol/L)	362.99 ± 94.60	334.95 ± 84.06	418.47 ± 89.66	<0.001
TG (mmol/L)	1.46 ± 1.05	1.10 ± 0.68	2.17 ± 1.28	<0.001
TC (mmol/L)	4.96 ± 0.91	4.89 ± 0.89	5.09 ± 0.94	<0.001
HDL (mmol/L)	1.40 ± 0.39	1.52 ± 0.38	1.16 ± 0.26	<0.001
LDL (mmol/L)	2.91 ± 0.78	2.84 ± 0.76	3.05 ± 0.80	<0.001
TSH (mIU/L)	2.01 ± 0.82	2.02 ± 0.82	1.99 ± 0.82	0.028
FT4 (pmol/L)	17.11 ± 2.02	17.14 ± 2.02	17.05 ± 2.01	0.005
FT3 (pmol/L)	5.06 ± 0.60	4.95 ± 0.59	5.28 ± 0.57	<0.001
FT3/FT4	0.30 ± 0.04	0.29 ± 0.04	0.31 ± 0.04	<0.001
TyG	8.51 ± 0.66	8.24 ± 0.55	9.03 ± 0.56	<0.001

MASLD, metabolic dysfunction-associated steatotic liver disease; BMI, body mass index; WC, waist circumference; SBP, systolic blood pressure; DBP, diastolic blood pressure; HB, hemoglobin; ALT, alanine aminotransferase; AST, aspartate aminotransferase; GGT, gamma-glutamyl transferase; FPG, fasting plasma glucose; UA, uric acid; TG, triglycerides; TC, total cholesterol; HDL, high-density lipoprotein; LDL, low-density lipoprotein; TSH, thyroid-stimulating hormone; FT3, free triiodothyronine; FT4, free thyroxine; TyG, triglyceride-glucose index.

Significant variations in thyroid function and TyG index were found between MASLD and non-MASLD groups. The MASLD group had a significantly higher mean FT3 level (5.28 ± 0.57 pmol/L) than the non-MASLD group (4.95 ± 0.59 pmol/L), *P* < 0.001. *A* lower mean TSH level (1.99 ± 0.82 mIU/L) was found in the non-MASLD group compared to the MASLD group (2.02 ± 0.82 mIU/L), *P* = 0.028. MASLD individuals had a slightly lower mean FT4 level (17.05 ± 2.01 pmol/L) than non-MASLD individuals (17.14 ± 2.02 pmol/L), *P* = 0.005. In the MASLD group, the mean TyG index was 9.03 ± 0.56, which was substantially higher than in the non-MASLD group (8.24 ± 0.55, *P* < 0.001).

### Correlation analysis between variables

**Figure 2:**
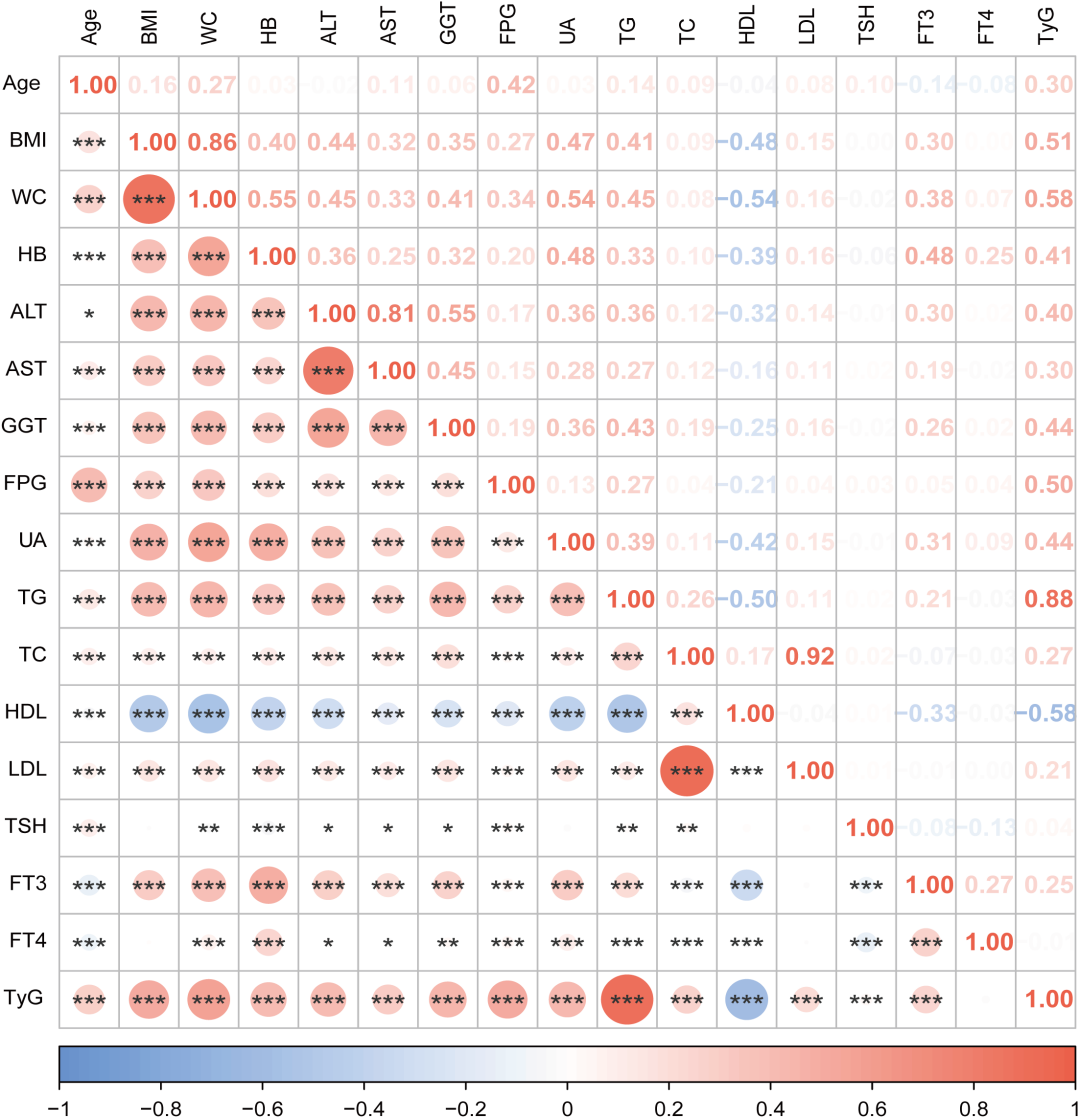
Correlation analysis between variables in this study. The size of the circle represents the strength of the correlation. Orange color for positive correlation, blue for negative correlation. *** indicates *P <*0.001, ** indicates *P* <0.01,* indicates *P <*0.05. BMI, body mass index; WC, waist circumference; HB, hemoglobin; ALT, alanine aminotransferase; AST, aspartate aminotransferase; GGT, gamma-glutamyl transferase; FPG,fasting plasma glucose; UA, uric acid; TG, triglycerides; TC, total cholesterol; HDL, high-density lipoprotein; LDL, low-density lipoprotein; TSH, thyroid-stimulating hormone; FT3, free triiodothyronine; FT4, free thyroxine; TyG, triglyceride-glucose index.

**Figure 2** illustrates the correlation between the variables. There was a positive correlation found between FT3 and TyG (r = 0.25, *P* < 0.001). FT3 and TyG exhibited positive correlations with a variety of metabolic parameters and liver enzymes, such as BMI, WC, total cholesterol (TC), low-density lipoprotein (LDL), aspartate aminotransferase (AST), UA, alanine aminotransferase (ALT) and gamma-glutamyl transferase (GGT). Conversely, they exhibited negative correlations with HDL.

### Logistic regression analysis of variables on the risk of MASLD

**Table 2** shows multiple metabolic factors associated with MASLD. We discovered that FT3 and TyG index increased MASLD risk in univariate logistic regression analyses. The odds ratio (OR) for FT3 was 2.68 (95% confidence interval (CI): 2.54-2.84; *P* < 0.001), while the TyG index on the risk of MASLD showed an OR of 12.25 (95% CI: 11.33-13.25; *P* < 0.001). After controlling for possible confounders including sex, age, hypertension, diabetes, BMI, WC, systolic blood pressure (SBP), diastolic blood pressure (DBP), HB, AST, ALT, GGT, UA, TC, HDL, LDL, TSH, and FT4, FT3 remained significantly associated with an increased risk of MASLD (OR = 1.35, 95% CI: 1.23-1.49; *P* < 0.001). Similarly, the TyG index was independently associated with higher MASLD risk (OR = 3.99, 95% CI: 3.40-4.68; *P* < 0.001).

**Table 2:** Univariate and multivariate logistic regression analysis of variables on the risk of MASLD.

Variable	Univariate logistic regression			Multiple logistic regression		
	OR	95% CI	*P* value	OR	95% CI	*P* value
Sex	4.43	4.12-4.77	<0.001	0.23	0.19-0.27	<0.001
Age	1.03	1.02-1.03	<0.001	1.02	1.01-1.02	<0.001
Hypertension	3.68	3.42-3.96	<0.001	1.24	1.09-1.41	0.001
Diabetes	3.78	3.39-4.21	<0.001	1.38	1.16-1.64	<0.001
BMI	1.70	1.67-1.72	<0.001	1.20	1.16-1.23	<0.001
WC	1.19	1.18-1.19	<0.001	1.08	1.07-1.09	<0.001
SBP	1.04	1.04-1.04	<0.001	0.99	0.99-1.00	0.005
DBP	1.07	1.07-1.07	<0.001	1.02	1.01-1.03	<0.001
HB	1.06	1.06-1.06	<0.001	1.01	1.01-1.02	<0.001
ALT	1.10	1.09-1.10	<0.001	1.06	1.06-1.07	<0.001
AST	1.12	1.11-1.13	<0.001	0.97	0.96-0.98	<0.001
GGT	1.04	1.04-1.05	<0.001	1.00	1.00-1.00	0.791
UA	1.01	1.01-1.01	<0.001	1.00	1.00-1.01	<0.001
TC	1.28	1.23-1.32	<0.001	0.55	0.44-0.69	<0.001
HDL	0.03	0.02-0.03	<0.001	0.51	0.39-0.66	<0.001
LDL	1.42	1.36-1.48	<0.001	2.02	1.60-2.55	<0.001
TSH	0.96	0.92-1.00	0.029	0.85	0.80-0.90	<0.001
FT4	0.98	0.96-0.99	0.005	0.93	0.91-0.95	<0.001
FT3	2.68	2.54-2.84	<0.001	1.35	1.23-1.49	<0.001
TyG	12.25	11.33-13.25	<0.001	3.99	3.40-4.68	<0.001

MASLD, metabolic dysfunction-associated steatotic liver disease; BMI, body mass index;WC,waist circumference; SBP, systolic blood pressure; DBP, diastolic blood pressure; HB, hemoglobin; ALT, alanine aminotransferase; AST, aspartate aminotransferase; GGT, gamma-glutamyl transferase; UA, uric acid; TC, total cholesterol; HDL, high-density lipoprotein; LDL, low-density lipoprotein; TSH, thyroid-stimulating hormone; FT3, free triiodothyronine; FT4, free thyroxine; TyG, triglyceride-glucose index.

Multiple logistic regression analysis revealed significant associations between MASLD and the following variables: sex, age, hypertension, diabetes, BMI, WC, SBP, DBP, HB, AST, ALT, UA, TC, HDL, LDL, TSH and FT4.

### Associations between FT3 and MASLD across subgroups

**Table 3** shows the results of the subgroup analysis to determine the associations between FT3 and MASLD. A significant positive association was observed between MASLD and FT3 overall (OR = 2.68, 95%CI: 2.54-2.84, *P* < 0.001). Subgroup analyses revealed significant interactions for age (*P* < 0.001), hypertension (*P* < 0.001), and TyG index (*P* < 0.001). The association was strongest in the 0–24 age group (OR = 5.5, 95% CI: 3.02-10.02) and gradually decreased with age, remaining significant in all age groups (all *P* < 0.001). The association was stronger in non-hypertensive individuals (OR = 3.15, 95% CI: 2.94-3.37) compared to hypertensive individuals (OR = 1.99, 95% CI: 1.78-2.23; both *P* < 0.001) and in those with a TyG index < 8.55 (OR = 2.93, 95% CI: 2.63-3.26) compared to TyG ≥ 8.55 (OR = 1.83, 95% CI: 1.69-1.98; both *P* < 0.001). No significant interactions were observed for BMI (*P =* 0.05) or sex (*P* = 0.896) or diabetes (*P* = 0.076).

**Table 3:** Association between FT3 and MASLD across subgroups.

Variable	n	OR	95% CI	*P* value	*P* for interaction
Overall	18298	2.68	2.54-2.84	<0.001	
Sex					0.896
Female	7399	1.81	1.61-2.04	<0.001	
Male	10899	1.83	1.70-1.97	<0.001	
Age					<0.001
0-24	358	5.5	3.02-10.02	<0.001	
25-44	9271	4.20	3.84-4.59	<0.001	
45-64	6856	2.38	2.17-2.61	<0.001	
≥ 65	1813	1.81	1.52-2.17	<0.001	
BMI					0.050
< 18.5	516	0.38	0.01-15.23	0.604	
18.5-23.9	8052	2.09	1.83-2.39	<0.001	
24-28	7002	1.70	1.56-1.86	<0.001	
≥ 28	2728	1.94	1.64-2.30	<0.001	
Hypertension					<0.001
No	14339	3.15	2.94-3.37	<0.001	
Yes	3959	1.99	1.78-2.23	<0.001	
Diabetes					0.076
No	16765	2.95	2.78-3.14	<0.001	
Yes	1533	2.45	2.02-2.98	<0.001	
TyG					<0.001
< 8.55	10059	2.93	2.63-3.26	<0.001	
≥ 8.55	8239	1.83	1.69-1.98	<0.001	

FT3, free triiodothyronine; MASLD, metabolic dysfunction-associated steatotic liver disease; BMI, body mass index; TyG, triglyceride-glucose index.

### Interaction between FT3 and TyG on the risk of MASLD

ROC curve analysis determined optimal FT3 and TyG index cutoffs for MASLD prediction based on the maximum Youden index. The calculated cutoff values were 8.55 for the TyG index and 4.98 for FT3. Based on these cutoffs, participants were categorized into four groups: low FT3 and low TyG, high FT3 and low TyG, low FT3 and high TyG, and high FT3 and high TyG.

The analysis of interaction effects between FT3 and TyG on MASLD risk revealed significant findings after multivariate adjustment (**Table 4**). Compared to the reference group (low FT3/low TyG), high FT3 alone (OR = 1.54, 95% CI: 1.31-1.81) and high TyG alone (OR = 4.20, 95% CI: 3.59-4.92) were both significantly associated with increased MASLD risk (both *P* < 0.001). The combination of high FT3 and high TyG demonstrated a synergistic effect, showing the highest risk elevation (OR = 5.38, 95% CI: 4.62-6.26, *P* < 0.001) and the highest MASLD prevalence (55.76% vs 5.38% in reference).

**Table 4:** Interaction between FT3 and TyG on the risk of MASLD.

High FT3	High TyG	non-MASLD n (%)	MASLD n (%)	OR	95% CI	*P*
–	–	5064 (41.66)	331 (5.38)	1.00	1.00	
**+**	–	3189 (26.24)	604 (9.83)	1.54	1.31-1.81	<0.001
–	**+**	1859 (15.29)	1783 (29.02)	4.20	3.59-4.92	<0.001
**+**	**+**	2042 (16.80)	3426 (55.76)	5.38	4.62-6.26	<0.001

Adjusted for age, sex, BMI, waist circumference, hypertension, diabetes, hemoglobin, aspartate aminotransferase, gamma-glutamyl transferase, uric acid. FT3, free triiodothyronine; TyG, triglyceride-glucose index; MASLD, metabolic dysfunction-associated steatotic liver disease.

We further calculated measures of additive interaction (RERI, AP, and SI) based on the OR-values from **Table 4**. The results showed RERI = 0.64 (95% CI: 0.06-1.21), AP = 0.12 (95% CI: 0.02-0.22), and SI = 1.17 (95% CI: 1.01-1.36). The positive values of both RERI and AP (> 0), along with SI > 1, indicate a statistically significant additive interaction between FT3 and TyG on MASLD risk.

## Discussion

According to our research, FT3 and TyG showed significant positive correlations with a range of metabolic parameters (BMI, WC, TC, LDL, UA) and hepatic enzymes (ALT, AST, and GGT) and negative correlations with HDL in a euthyroid population. An increased risk of MASLD was shown to be related with the FT3 and TyG index. Compared to the reference group (low FT3/low TyG), the combination of high FT3 (≥ 4.98 pmol/L) and high TyG (≥ 8.55) exhibited significantly elevated MASLD risk (OR = 5.38, 95% CI: 4.62-6.26; *P* < 0.001).

We confirmed a significant association between elevated FT3 levels and increased MASLD risk. Subgroup analysis indicated that the association between FT3 and MASLD risk was more pronounced in specific subgroups, including younger individuals, those without hypertension, and those with lower TyG index. After controlling for known confounders, this association remained significant. These observational data demonstrate a correlation between FT3 levels and MASLD presence, particularly in individuals with less severe metabolic dysfunction. It is still not completely known what causes the levels of FT3 to rise. Insulin resistance is common in MASLD patients, and this might cause a comparatively high level of intracellular T3 synthesis as a compensatory mechanism for the local tissue’s altered metabolic status (28, 29). The goal of increasing local T3 synthesis might be to simulate metabolic activity and induce tissue thermogenesis (29, 30). There may be an increase in FT3 levels inside the liver tissue as a result of thyroid hormone resistance manifesting primarily in the liver in MASLD (31, 32).

The TyG index was found to have a significant association with the risk of MASLD, as demonstrated by our findings. Our findings confirm and extend previous observations by Zeng et al. regarding the TyG index-NAFLD/MASLD relationship (22). Their study in a non-diabetic population demonstrated that TyG predicts NAFLD risk, particularly when combined with BMI (TyG-BMI) in non-obese individuals (22). There is also a substantial association between TyG and other metabolic illnesses, such as insulin resistance (23), type 2 diabetes (33, 34), and cardiovascular disease (35, 36), according to investigations that have been conducted.

Our research shows that the TyG index and FT3 interact significantly to affect the risk of MASLD. This discovery points to a synergistic impact, whereby increased FT3 and TyG levels greatly increase the risk of MASLD, much more than their separate effects. Increased levels of FT3 can result in oxidative stress by promoting metabolic rate and the generation of reactive oxygen species (ROS) (10). Oxidative stress causes harm to cellular structures, including those in the liver, resulting in damage to liver cells and the accumulation of fat in the liver steatosis (37). In addition, FT3 promotes hepatic gluconeogenesis by upregulating PEPCK/G6Pase (12), thereby exacerbating insulin resistance. A high TyG index signifies the presence of insulin resistance (22), which is linked to persistent low-level inflammation (38). FT3-induced ROS exacerbate TyG-related inflammation. The combined impact of inflammation and oxidative stress can lead to liver damage by triggering hepatic steatosis, inflammation, and fibrosis, which are the primary characteristics of MASLD (37).

The findings of our research have important clinical significance. Initially, it is emphasized that it is important to take into account FT3 levels, even if they fall within the normal range, when evaluating the risk of MASLD. This is especially crucial when considering other metabolic risk factors, such as those indicated by the TyG index. Furthermore, the results of our study highlight the significance of monitoring both the FT3 and the TyG index in order to identify people who are at a greater likelihood for MASLD. This will allow for prompt interventions and individualized treatment approaches.

Several important considerations must be made while addressing the study’s limitations: 1. Our research is limited in its ability to infer causality due to the cross-sectional study design. Future longitudinal studies are needed to investigate the potential causal roles of FT3 and the TyG index in the progression of MASLD. 2. individuals’ dietary habits and level of physical activity are not well documented, which is a major drawback because these factors are known to affect the risk of MASLD. 3. Even after controlling for known confounders, there may be some remaining confounding factors that were either not measured or were not corrected enough, and they could still affect the results of the study. 4. Our study used standardized ultrasonography for MASLD diagnosis but lacked fibrosis biomarkers such as FIB-4 for prognostic stratification. Future studies incorporating these non-invasive scores would better characterize disease severity.

In conclusion, elevated FT3 and TyG index are independently associated with an increased risk of MASLD, and they exhibit a significant synergistic additive interaction.

## Data Availability

The data analyzed in this study is subject to the following licenses/restrictions: The datasets used and/or analysed during the current study are available from the corresponding author on reasonable request. Requests to access these datasets should be directed to huyun304@njglyy.com.
