# Supplementary material for: Free triiodothyronine and triglyceride-glucose index interaction on metabolic dysfunction-associated steatotic liver disease risk in euthyroid individuals
Source: Front Endocrinol (Lausanne). 2025 Apr 24;16:1526198. doi: 10.3389/fendo.2025.1526198 (PMC12058485; doi:10.3389/fendo.2025.1526198)
Supplement: Supplementary file 1 [file Table1.docx]

**Supplementary Table 1** Multiplicative Interaction Analysis Between FT3 and Other Variables in Predicting MASLD

|  | B | SE | Z | *P* |
| --- | --- | --- | --- | --- |
| (Intercept) | -4.514 | 0.296 | -15.225 | 0.000 |
| FT3 | 0.596 | 0.061 | 9.770 | 0.000 |
| Sex1 | 1.135 | 0.356 | 3.191 | 0.001 |
| FT3*Sex1 | 0.009 | 0.071 | 0.131 | 0.896 |
|  |  |  |  |  |
| (Intercept) | -11.275 | 0.524 | -21.501 | 0.000 |
| FT3 | 1.752 | 0.101 | 17.354 | 0.000 |
| Age | 0.100 | 0.011 | 9.495 | 0.000 |
| FT3*Age | -0.013 | 0.002 | -6.162 | 0.000 |
|  |  |  |  |  |
| (Intercept) | -16.881 | 1.873 | -9.011 | 0.000 |
| FT3 | 0.681 | 0.365 | 1.865 | 0.062 |
| BMI | 0.537 | 0.074 | 7.275 | 0.000 |
| FT3*BMI | -0.006 | 0.014 | -0.389 | 0.697 |
|  |  |  |  |  |
| (Intercept) | -6.887 | 0.184 | -37.379 | 0.000 |
| FT3 | 1.147 | 0.035 | 32.629 | 0.000 |
| Hypertension1 | 3.689 | 0.348 | 10.590 | 0.000 |
| FT3*HypertensionT1 | -0.457 | 0.068 | -6.735 | 0.000 |
|  |  |  |  |  |
| (Intercept) | -6.362 | 0.163 | -39.091 | 0.000 |
| FT3 | 1.082 | 0.031 | 34.744 | 0.000 |
| Diabetes1 | 2.458 | 0.516 | 4.761 | 0.000 |
| FT3*Diabetes1 | -0.185 | 0.104 | -1.774 | 0.076 |
|  |  |  |  |  |
| (Intercept) | -34.609 | 3.180 | -10.882 | 0.000 |
| FT3 | 2.544 | 0.614 | 4.141 | 0.000 |
| TyG | 3.496 | 0.366 | 9.560 | 0.000 |
| FT3*TyG | -0.210 | 0.071 | -2.967 | 0.003 |
